# Nuclear shell-model simulation in digital quantum computers

**DOI:** 10.1038/s41598-023-39263-7

**Published:** 2023-07-29

**Authors:** A. Pérez-Obiol, A. M. Romero, J. Menéndez, A. Rios, A. García-Sáez, B. Juliá-Díaz

**Affiliations:** 1 Barcelona Supercomputing Center, 08034 Barcelona, Spain; 2 Departament de Física Quàntica i Astrofísica (FQA), Universitat de Barcelona (UB), c. Martí i Franqués, 1, 08028 Barcelona, Spain; 3 Institut de Ciències del Cosmos (ICCUB), Universitat de Barcelona (UB), c. Martí i Franqués, 1, 08028 Barcelona, Spain; 4 Qilimanjaro Quantum Tech, 08007 Barcelona, Spain

**Keywords:** Nuclear physics, Quantum physics

## Abstract

The nuclear shell model is one of the prime many-body methods to study the structure of atomic nuclei, but it is hampered by an exponential scaling on the basis size as the number of particles increases. We present a shell-model quantum circuit design strategy to find nuclear ground states by exploiting an adaptive variational quantum eigensolver algorithm. Our circuit implementation is in excellent agreement with classical shell-model simulations for a dozen of light and medium-mass nuclei, including neon and calcium isotopes. We quantify the circuit depth, width and number of gates to encode realistic shell-model wavefunctions. Our strategy also addresses explicitly energy measurements and the required number of circuits to perform them. Our simulated circuits approach the benchmark results exponentially with a polynomial scaling in quantum resources for each nucleus. This work paves the way for quantum computing shell-model studies across the nuclear chart and our quantum resource quantification may be used in configuration-interaction calculations of other fermionic systems.

## Introduction

Atomic nuclei are complex many-body systems formed by protons and neutrons (collectively denoted as nucleons) bound by the strong nuclear force. Nuclei exhibit captivating properties such as the coexistence of spherical and deformed shapes at low energies^1–3^, strong short-range correlations between pairs of nucleons^4^, or decay modes driven by the strong^5^, weak^6^ or electromagnetic^7^ forces. Furthermore, nuclear decays are crucial to understand the origin of heavy elements in the universe^8^, and experiments using nuclei aim to answer fundamental physics questions such as which is the nature of dark matter^9^, why matter dominates over antimatter in the universe^10^, or whether neutrinos are their own antiparticles^11^.

The nuclear shell model, also known as the configuration-interaction method, is one of the leading many-body approaches to study the structure of nuclei. The shell model is grounded in the idea that, in a similar fashion to electrons in an atom, nucleons occupy orbitals organized in *shells* of different energies^12,13^. Nuclear states are then obtained by computationally intensive diagonalizations of the nuclear Hamiltonian in a many-body configuration space comprising one or several shells. In spite of impressive progress in recent decades^14–17^, the exponential scaling of the many-body Hilbert space with the number of nucleons ultimately prevents the application of the shell model across the entire nuclear chart, particularly in heavy nuclei.

Quantum computing promises to circumvent limitations associated to any exponentially-scaling many-body system using the principle of superposition of qubit states^18^. In the current noisy intermediate-scale quantum (NISQ) device era^19^, *variational quantum eigensolvers* (VQE)^20,21^ are among the most successful algorithms^22^ exploiting the benefits of quantum computing to deal with complex many-body problems in physics^23,24^ and chemistry^25–27^. Quantum many-body systems that have been used as VQE testbeds include the Fermi-Hubbard^28^, Ising^29^ and Lipkin–Meshkov–Glick models^30–34^, superfluid systems^35,36^, hadrons^37^ or molecules^38–40^.

**Figure 1:**
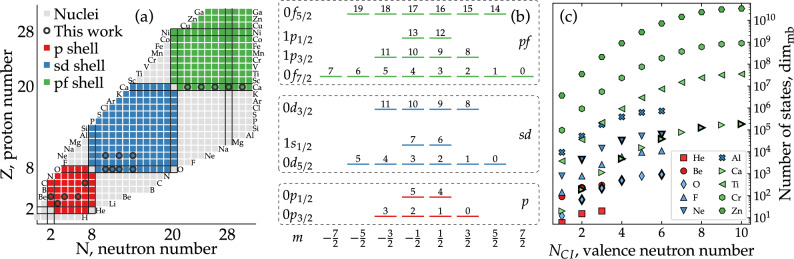
The shell model and quantum encoding. (**a**) Segrè chart covering the *p*, *sd* and part of the *pf* shell. Solid lines indicate neutron and proton magic numbers. Open circles show the isotopes studied in this work. (**b**) Schematic representation of the *p*-, *sd*- and *pf*-shell configuration spaces. The number on top of every single-particle state is the qubit label for the implementation in a quantum device under a Jordan–Wigner mapping. (**c**) Number of many-body configurations, $\dim _\mathrm{{mb}}$, in the *M*-basis as a function of the number of active neutrons in the configuration space, $N_\mathrm{{CI}}$. We show results for the isotopic chains of He and Be in the *p* shell; O, F, Ne, and Al in the *sd* shell; and Ca, Ti, Cr, and Zn in the *pf* shell. Isotopes beyond the middle of the shell are not shown since the number of configurations is symmetric. Bold marker lines highlight nuclei studied in this work.

In general, a VQE implementation requires a series of well-defined stages^24^, involving (a) a mapping between physical degrees of freedom (eg fermionic operators) and the qubits in a quantum computer; (b) the preparation of an initial reference state; (c) a (potentially iterative) variational optimization; (d) a measurement strategy for expectation values of operators (most importantly, the Hamiltonian); and (e) an error mitigation scheme. Previous nuclear shell-model studies have only partially tackled these problems^41–44^. The aim of this article is to present a circuit design strategy that explicitly addresses all these aspects to solve the nuclear shell model in a quantum computer. We also quantify the necessary circuit resources, such as depths and widths, to achieve precise predictions for nuclear masses. We do this in a set of test nuclei across different nuclear shells. To this end, we perform (classical) baseline simulations on the corresponding circuit architectures and benchmark the results against diagonalizable shell-model simulations as well as independent ADAPT-VQE simulations without an explicit circuit implementation.

## Results

### Nuclear shell model

The nuclear shell model^14–17^ considers nuclei composed by an inert core of nucleons, which do not explicitly contribute to the dynamics, and a set of valence protons and neutrons interacting in a relatively small configuration space. This space is usually bounded by two *magic numbers*, which denote special configurations of protons or neutrons leading to particularly stable nuclei. Magic numbers thus define shells with large energy gaps between them. Configuration spaces used in shell-model calculations usually comprise one or two shells. Panel (a) of Fig. 1 shows the light to mid-mass region of the isotope chart. We highlight areas where the *p*, *sd* and *pf* shell-model calculations are routinely employed.

Since the nuclear force is rotationally invariant and nucleons are fermions, it is useful to work in a single-particle basis with states with quantum numbers $n\,l_{j}$, where *n* is the principal quantum number, *l* the orbital angular momentum and *j* the total angular momentum^45^. This basis also includes *m* third-component projections of *j* degenerate in energy. The nuclear Hamiltonian is also to a very good approximation the same for neutrons and protons, so it is customary to define, additionally, the isospin quantum number $t=1/2$, with third component $t_z$ discerning protons and neutrons^46^. Many-body nuclear states have good total angular momentum *J* and isospin *T*, with respective third components *M* and $T_z$ given by the sum of the third components of all nucleons in the nucleus^47^.

The nuclear Hamiltonian in a given configuration space can be written as

1 $$\begin{aligned} H_\mathrm{{eff}} = \sum _i \varepsilon _i a_i^{\dag } a_i + \frac{1}{4} \sum _{ijkl}{\bar{v}}_{ijkl} a_i^{\dag } a_j^{\dag } a_l a_k, \end{aligned}$$

where $\varepsilon _i$ is the energy of the single-particle state *i* and ${\bar{v}}_{ijkl} = v_{ijkl} - v_{ijlk}$ are antisymmetrized two-body matrix elements. $a_i$ and $a_i^{\dag }$ are fermionic annihilation and creation operators associated to each single-particle state, *i*. The matrix elements ${\bar{v}}_{ijkl}$ can be obtained^17,48^ from an effective field theory of the underlying theory of the nuclear force, quantum chromodynamics^49^. Here, instead, we use standard phenomenological Hamiltonians, with components adjusted to better reproduce key properties of selected nuclei^50^. We choose the Cohen–Kurath interaction in the *p* shell^51^, USDB in the *sd* shell^52^ and KB3G in the *pf* shell^53^.

A suitable many-body basis, also referred to as Fock space, for shell-model calculations is provided by the so-called $M-$scheme^46^, in which the Slater determinant states are chosen to have a well-defined *M*. $T_z=(N-Z)/2$ is also well defined because the number of neutrons *N* and protons *Z* is fixed. Nuclear states are thus expanded in this basis,

2 $$\begin{aligned} | JM\,TT_z\rangle = \sum _{\alpha } c_{\alpha } | \alpha , M T_z\rangle , \end{aligned}$$

and nuclear wavefunctions and their corresponding energies are eigenvectors and eigenvalues of the Hamiltonian matrix in the basis of Slater determinants. The $c_\alpha$ coefficients are obtained through diagonalization employing state-of-the-art nuclear shell-model codes^54–57^ and ensure that eigenstates have good *J* and *T* quantum numbers.

However, this framework faces a steep computational bottleneck in terms of the maximum size of the Hamiltonian matrix from which the lowest eigenvalues and eigenvectors can be calculated. The dimension of the single-particle basis of a nuclear shell consisting of several orbitals $nl_j$ is

3 $$\begin{aligned} \dim _\mathrm{{sp}} = \sum _j (2j+1), \end{aligned}$$

where the sum runs over the *j* values in a given configuration space, see panel (b) of Fig. 1 for details. The corresponding number of Slater determinants grows combinatorially as

4 $$\begin{aligned} \dim _{\text {mb}} = \left( {\begin{array}{c}\dim _{\text {sp}}\\ N_{\text {CI}}\end{array}}\right) \times \left( {\begin{array}{c}\dim _{\text {sp}}\\ Z_{\text {CI}}\end{array}}\right) , \end{aligned}$$

where $N_{\text {CI}}$ ($Z_{\text {CI}}$) is the number of active neutrons (protons) in the configuration space. Let us consider the *sd* shell, comprising the $1s_{1/2}$, $0d_{3/2}$ and $0d_{5/2}$ orbitals for both protons and neutrons, and the *pf* shell, comprising the $0f_{7/2}$, $0f_{5/2}$, $1p_{3/2}$ and $1p_{1/2}$ orbitals. There are 12 (20) single-particle states in the *sd* (*pf*) shell, so that it can describe the isotopic chains of 12 (20) elements with up to 12 (20) valence neutrons, as shown in panel (a) of Fig. 1. Panel (c) illustrates the exponential scaling of the number of many-body configurations, $\dim _\mathrm{{mb}}$, present for isotopes of elements in different shells. The number of basis states needed to describe two isotopes of the same element, or two elements with the same *N* in the same shell, can differ by three or more orders of magnitude. In practical calculations, this number may be reduced by about an order of magnitude due to symmetry considerations, leading to a reduced number of Slater determinants, $N_{\text {SD}}$^15^. However, the scaling in either $\dim _{\text {mb}}$ or $N_{\text {SD}}$ ultimately places a limit in the computational resources needed to study heavy nuclei with the nuclear shell model. This refers to both the number of operations per second, or CPU time, and the memory to store all configurations. In fact, the shell-model history is closely tied to that of computation, as larger-scale calculations became feasible with the advances in computational power and refined techniques in CPUs and GPUs^14–17^.

### Variational algorithm

Here, we implement the nuclear shell model in a quantum computer following a standard Jordan–Wigner (JW) mapping^43,44,58,59^. We associate each qubit with a single-particle state in the configuration space, which can either be empty (projection 0) or occupied (projection 1). Panel (b) of Fig. 1 shows the mapping between single-particle states and qubits for the *p* (bottom), *sd* (central) and *pf* shells (top panel). From a memory-storage perspective, a shell-model VQE under the JW mapping only requires as many qubits as single-particle states in the configuration space. In other words, the number of qubits remains constant for all nuclei described within a given shell. If a VQE can be used to diagonalize the problem and is robust against errors, the approach may provide access to much larger configuration spaces, currently unattainable in classical computers.

A VQE uses the Rayleigh–Ritz variational principle^60,61^ to calculate the ground-state of a Hamiltonian starting from an initial ansatz. Our algorithm of choice is ADAPT-VQE^27,38,40,59,62^, which iteratively builds a wavefunction of the form

5 $$\begin{aligned} |\psi (\varvec{\theta })\rangle = \prod _{k=1}^n e^{i\theta _k A_k} |\text{ref}\rangle , \end{aligned}$$

where $|\text{ref}\rangle$ is an initial (reference) state of the quantum system, *k* is the iteration (or layer) index, $A_k$ are particle-hole excitation operators, and $\varvec{\theta }=\{ \theta _i, i=1,\dots , n \}$ are a set of variational parameters. We stress that the adapted wavefunction in Eq. (5) is free of Trotter–Suzuki approximation errors^63,64^. This ansatz does not require decomposing an exponential map of a sum of excitation operators, as would be the case in algorithms such as UCC-VQE^25,44^.

The minimization of the energy of this wavefunction with respect to the parameters $\varvec{\theta }$,

6 $$\begin{aligned} E = \min _{\varvec{\theta }} \frac{\langle \psi (\varvec{\theta }) | H_\mathrm{{eff}} |\psi (\varvec{\theta })\rangle }{\langle \psi (\varvec{\theta }) |\psi (\varvec{\theta })\rangle }, \end{aligned}$$

can be performed classically^65^ and yields an approximate ground-state energy. Here, we use the BFGS optimizer with a gradient tolerance set to $10^{-6}$ at every iteration. At each layer *k* of the iterative procedure, the ansatz grows by one parametrized unitary, $|\psi (\varvec{\theta })\rangle \rightarrow e^{i\theta _k A_k}|\psi (\varvec{\theta })\rangle$. The new operator $A_k$ is selected according to the largest energy gradient computed as

7 $$\begin{aligned} \left. \frac{\partial E^{(n)}}{\partial \theta _k } \right| _{\theta _k=0} = \left. i \langle \psi (\varvec{\theta })| [H_\mathrm{{eff}},A_k] |\psi (\varvec{\theta })\rangle \right| _{\theta _k=0}. \end{aligned}$$

Thus, at every layer, the wavefunction adapts to the new information acquired in the previous optimization. The set of parameters $\varvec{\theta }$ are obtained anew for every layer, so an updated state has no ties to former states. The adaptive character of ADAPT-VQE should lead to implementations with shallower circuits^38,40^.

A crucial point for the optimal convergence towards the target state is the choice of excitation operators $A_k$. These are predefined in an operator pool, prior to the start of the simulation. Since our interest lies in the nuclear shell model, with a Hamiltonian of the form in Eq. (1), we use a pool of two-body fermionic excitation operators

8 $$\begin{aligned} T_{rs}^{pq} = i (a_p^{\dag } a_q^{\dag } a_r a_s - a_r^{\dag } a_s^{\dag } a_p a_q), \end{aligned}$$

where *p*, *q*, *r* and *s* are single-particle labels with quantum numbers *n*, *l*, *j*, *m* and $t_z$. The same operator may be selected more than once throughout the iterative process, but not on consecutive iterations. We apply symmetry considerations when building the Slater determinant basis for the nuclear ground state, and only consider excitation operators which conserve the total angular momentum and isospin projection *M* and $T_z$. This iterative procedure continues until convergence, defined when all the gradient norms in Eq. (7) vanish and/or when the energy is close enough to a known solution from, for instance, classical diagonalization benchmarks. While one could consider more complex operators, involving triple or quadruple particle-hole excitations^43,44^, our simulations indicate that, for the wide set of nuclei studied in this work, full shell-model correlations can be captured at the two-body level with a commensurate number of ansatz layers, of at most a few hundred.

### Circuit design strategy

The main aim of this paper is to determine the optimal architecture of quantum circuits that can implement a nuclear shell-model VQE. We explore all the necessary stages of a VQE, from the encoding to the energy measurement in the “Methods” section. Ultimately, the circuit design strategy that we propose provides an approximation-free implementation of ADAPT-VQE, in a one-to-one correspondence with the method^38,62^. Having access to the circuit structure across the full VQE minimization process, including energy measurements, is a key step forward in discussing the scalability of nuclear shell-model simulations in quantum devices, and it is particularly critical to estimate the necessary resources for nuclear shell-model simulations with a real quantum advantage, that is, in isotopes or regions of the chart where current classical devices cannot be employed.

We benchmark our circuit implementation with circuit-free ADAPT-VQE simulations^59^. The latter implement the full algorithm using regular matrix calculus, expressing statevectors, Hamiltonians and pool operators as sparse matrices in the Fock basis. With the circuit for the ansatz built and optimized, we simulate the energy measurement protocol, to test the circuits for the changes of basis needed to extract energies in an actual quantum computer.

The state preparation protocol is the most resource-intensive part of the algorithm and we provide indications of the resource costs in the Simulations subsection. We can also quantify and optimize the scaling of the energy measurements. The nuclear shell-model Hamiltonian in Eq. (1) consists of one and two-body operators, which can be expressed in terms of Pauli strings (see the “Methods” section). The one-body part of the Hamiltonian is diagonal and can be measured directly. We divide the two-body part in three different kinds of terms, depending on the number of repeated indices. Table 1 lists the number of circuits needed to measure the expectation value of each part of the Hamiltonian for the *p*, *sd* and *pf* shells. Our design strategy indicates that 100 circuits should suffice to compute any isotope in the *p* shell and semi-magic nuclei in the *sd* shell. Open-shell isotopes require a factor of 4–6 more circuits than their semi-magic counterparts in a given shell.

In a quantum computer implementation, an energy calculation will be affected by statistical errors. Across a whole ADAPT-VQE simulation, the total number of circuits to be measured for each layer will be the product of three terms, $N_s\times N_{tot}\times N_{fc}$. The number of shots, $N_s$, is of statistical nature and, as discussed in the Methods section in the context of Eq. (19), it will be sensitive to error mitigation schemes.

$N_{tot}$ is the number of different energy measurement circuits. We estimate this number and show the results in Table 1. Finally, $N_{fc}$ is the number of function calls from the classical optimizer, which we analyze in the Supplementary Information.

**Table 1:** Number of circuits needed to measure the expectation value of the nuclear shell-model for the *p*, *sd* and *pf* shells.

Shell	$N_{qb}$	$N_\text {h}$	$N_{\text {hh}}$	$N_{tot}$
*p*	6	2	10 (9)	13 (12)
	12	4	109 (44)	114 (49)
*sd*	12	8	203 (86)	212 (95)
	24	16	1389 (518)	1406 (535)
*pf*	20	20	1507 (570)	1528 (591)
	40	40	10,572 (3459)	10,613 (3500)

$N_{qb}$ indicates the number of qubits for only neutrons or protons (top row for each shell) or both nucleon types (bottom). $N_{\text {h}}$ and $N_{\text {hh}}$ are the number of single- and double-hopping terms in the Hamiltonian (related to $h_{ijki}$ and $h_{ijkl}$, respectively), defining the number of circuits needed to measure these parts. The last column lists the total number of circuits, $N_{\text {h}}+N_{\text {hh}}+1$, accounting also for the single circuit needed to measure $\langle n_i\rangle$ and $\langle h_{ijij}^{(l)}\rangle$. The values in parenthesis correspond to the minimum number of groups containing $h_{ijkl}$ terms that commute with each other and thus can be measured with the same circuit.

### Simulations

**Table 2:** Ansatz and circuit depth for a given energy bound.

Shell	$N_{qb}$	$N_{\text {SD}}$	Nucleus	$N_{\text{layers}}$	$\varepsilon _E$ bound	$N_{\text{C}}$ (bound)
*p*	6	5	$^6{}$Be	2	$10^{-8}$	42 (80)
	12	10	$^6{}$Li	9	$10^{-7}$	92 (176)
		53	$^8{}$Be	48	$10^{-7}$	68 (176)
		51	$^{10}{}$Be	48	$10^{-7}$	62 (176)
		21	$^{13}{}$C	19	$10^{-7}$	77 (176)
*sd*	12	14	$^{18}{}$O	5	$10^{-6}$	99 (176)
		37	$^{19}{}$O	32	$10^{-6}$	85 (176)
		81	$^{20}{}$O	70	$10^{-6}$	98 (176)
		142	$^{22}{}$O	117	$10^{-6}$	93 (176)
	24	640	$^{20}{}$Ne	167	$2\times 10^{-2}$	137 (368)
		4206	$^{22}{}$Ne	236	$2\times 10^{-2}$	137 (368)
		7562	$^{24}{}$Ne	345	$2\times 10^{-2}$	138 (368)
*pf*	20	30	$^{42}{}$Ca	9	$10^{-8}$	116 (304)
		565	$^{44}{}$Ca	132	$10^{-2}$	153 (304)
		3952	$^{46}{}$Ca	124	$10^{-2}$	139 (304)
		12,022	$^{48}{}$Ca	101	$10^{-2}$	137 (304)
		17,276	$^{50}{}$Ca	221	$10^{-2}$	130 (304)

Number of ansatz layers ($N_{\text{layers}}$) and relative-error ($\varepsilon _E$) upper bounds for the ground-state energy of all nuclei simulated in this work, organized according to their configuration space (*p*, *sd*, and *pf* shells), number of qubits $N_{qb}$, and of many-body configurations (Slater determinants) $N_{SD}$. The last column reports the average number of CNOT gates per layer $N_\text{C}$ together with its upper bound, $16(N_{qb}-2)$ (see “Methods” section). For nuclei with $N_{\text{layers}}>100$, the average only accounts for the first 100 layers.

The systems we explore include nuclei across different shells, with even and odd numbers of protons and neutrons (see panel (a) of Fig. 1). We find that circuit-free and circuit-full simulations employing the same parameter minimization algorithm agree to numerical accuracy. We estimate the required depth of a circuit by imposing bounds on the relative error of the ground-state energy, $\displaystyle \varepsilon _E= \frac{|E-E_{\text {SM}} |}{E_{\text {SM}}}$, where $E_{\text {SM}}$ is the corresponding classical shell-model diagonalization result. Table 2 lists the number of ADAPT-VQE layers needed in an ansatz state to achieve a given value of $\varepsilon _E$ for a series of nuclei across the *p*, *sd* and *pf* shells. All energies tend to converge to the benchmark values, albeit with different rates. Semi-magic nuclei close to the closed shell typically converge rapidly, with less than 10 ADAPT-VQE layers. In contrast, the most costly nuclei simulated in this work, neon isotopes, require a few hundred ADAPT-VQE layers to reach a ground-state energy error of 2%. Nonetheless, we stress that the optimizations do not get stuck in barren plateaus. A key advantage of our circuit design strategy is that it allows us to quantify the associated quantum circuit resources. We take the number of CNOT gates required in the state preparation, $N_\text {CNOT}$, as a quantitative indicator of circuit resources.

**Figure 2:**
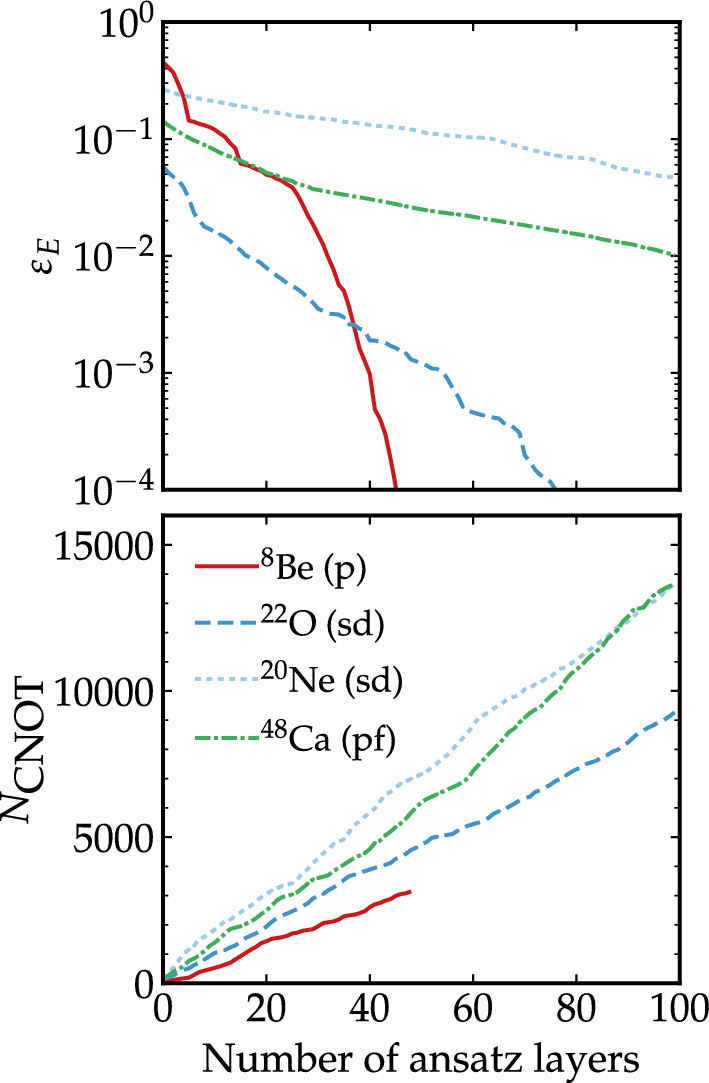
Energy relative error and circuit complexity as a function of ADAPT-VQE layers. Evolution of the relative error for the ground-state energy, $\varepsilon_\text{E}$, (top panel) and number of CNOT gates in the ansatz circuit (bottom) as a function of the number of ansatz layers for simulations of $^{8}$Be, $^{22}$O, $^{20}$Ne and $^{48}$Ca. As the algorithm adaptively iterates, errors decay exponentially while the number of CNOT gates increases linearly or polynomially.

Figure 2 shows the evolution of $\varepsilon _E$ (top panel) and $N_\text {CNOT}$ (bottom) as a function of the number of ADAPT-VQE layers for four representative isotopes across different nuclear shells. Simulations for all nuclei show that $\varepsilon _E$ decreases exponentially as the number of layers in the ansatz increases, while the number of CNOT gates grows linearly or polynomially. This number depends on the particular operators chosen by the ADAPT-VQE minimization, but it is at most $16\,(N_{qb}-1)$ per ansatz layer (see “Methods” section). In contrast, the average number of CNOT gates per ansatz layer found by ADAPT-VQE simulations is roughly half of the corresponding upper bounds, see Table 2. As an example, finding the ground-state energy of $^{22}$O with an error of few percent, requires about 20 ansatz layers and $\approx 2000$ CNOT gates. We provide more details for all the nuclei studied in this work in the Supplementary Information.

Figure 2 and Table 2 demonstrate that ADAPT-VQE converges exponentially as the number of layers, or equivalently CNOT gates, is increased.

**Figure 3:**
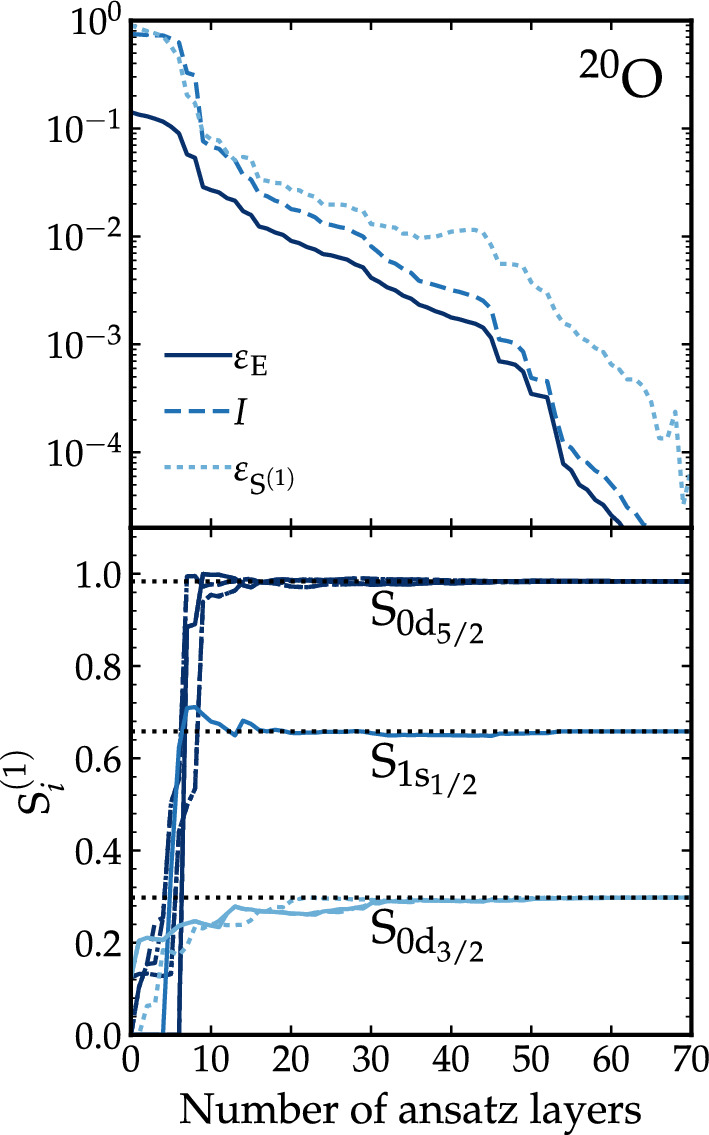
Quality of the wavefunction and entanglement entropy as a function of ADAPT-VQE layers. Evolution of the relative error for the ground-state energy, $\varepsilon _\text{E}$, the infidelity, *I*, and the average relative error of single-orbital entropies, $\varepsilon _{S^{(1)}}$ for $^{20}$O as a function of the number of ansatz layers (top panel). Evolution of $S_i^{(1)}$ for the same nucleus and *i* orbitals $0d_{3/2}$, $1s_{1/2}$ and $0d_{5/2}$, where the dotted lines indicate the entropies for the exact solution (bottom panel). The maximum $S_k^{(1)}$ is 1, very close to the value of the $0d_{5/2}$ orbitals.

Our results are either commensurate or competitive compared to previous estimates of circuit depth based on UCC-VQE on the *p* shell and on two oxygen isotopes on the *sd* shell^43,44^. For $^8$Be, Stetcu et al. require 112 variational parameters to reach $\varepsilon _E \approx 1 \%$ even after including triple and quadruple excitation operators^43^. Our implementation of ADAPT-VQE, with two-body excitation operators only, requires 48 parameters to reach $\varepsilon _E=10^{-7}$. In $^{22}$O, the UCC-VQE ansatz leads to $\varepsilon _E \approx 3 \%$ with 35 parameters^43^, whereas Fig. 3 indicates that ADAPT-VQE reaches a similar level of accuracy with about 20 layers. For $^6$Li, we find that 9 layers suffice to get a converged result up to $10^{-7}$, in contrast to the observations of Ref.^44^, where an alternative ADAPT-VQE implementation reaches only $\varepsilon _E \approx 10^{-3}$. A difference between previous implementations and our work is that we let our classical minimizer reach bottom precision at each ADAPT-VQE layer, whereas Kiss et al. employ 10 minimization steps per layer (with the SPSA optimizer)^44^. Moreover, UCC-VQE shell-model implementations have so far relied on Hartree–Fock reference states, which may not be optimal starting points for VQEs^59,66^. Either way, it appears that ADAPT-VQE shell-model simulations outperform their UCC-VQE counterparts in terms of layers, an observation that is in line with findings in quantum chemistry^27^. We note, however, that an unbiased comparison of quantum hardware efficiency between different methods requires a one-to-one quantification of the resources in each approach, including explicitly energy measurement overheads.

ADAPT-VQE predicts the ground-state energy of the nucleus, but one also has access to the nuclear wavefunction $|\psi (\varvec{\theta })\rangle$, although reconstructing it from quantum hardware may require costly quantum tomography. One can quantify the quality with respect to a given benchmark wavefunction, $|\psi _\text {b} \rangle$, by employing the infidelity $I= 1-|\langle \psi _\text {b}|\psi (\varvec{\theta }) \rangle |^2$. We take the classical shell model as a benchmark, and the better the level of agreement between both wavefunctions, the closer *I* is to 0. We also use the single-orbital entanglement entropy, $S_{i} = -(1-\gamma _{i}) \log _2 (1-\gamma _{i}) -\gamma _{i} \log _2 \gamma _{i}$, with $\gamma _{i}= \langle \psi (\varvec{\theta }) |a^\dagger _i a_i |\psi (\varvec{\theta }) \rangle$ , bound between 0 and 1, to evaluate the importance of quantum correlations in the ansatz^67–72^. These two indicators provide quantitative complementary information on the quality of the wavefunction and the variational process. Focusing on the test case example of $^{20}$O, the top panel of Fig. 3 shows the infidelity *I* of the ground state with respect to the shell-model wavefunction (dashed line). The panel also shows the average of relative errors of each single-particle state entanglement entropy, $\varepsilon _{S^{(1)}}=\frac{1}{N_{qb}}\sum _i \varepsilon _{S_i^{(1)}}$ (dotted line). These two quantities follow closely $\varepsilon _E$ along the iterative process. We observe a few sudden drops in the relative error for the energy, which correlate with similar drops in *I* and $\varepsilon _{S^{(1)}}$. This indicates that, at certain points in the optimization, ADAPT-VQE entangles parts of the nucleus relatively faster than others. Overall, the curves suggest that the ADAPT-VQE ansatz captures efficiently the entanglement structure of the many-body wavefunction. A more extensive analysis of the infidelity is provided in the Supplementary Information. The bottom panel of Fig. 3 provides a closer inspection to the entanglement structure of this nucleus. Based on previous studies^43,68,69^, we expect nuclear-structure features to correlate with single-particle states entanglement properties. The panel shows the quantum simulated single-orbital entropies of the 12 single-particle states as a function of the number of ansatz layers, compared to the classical shell-model entropies (horizontal dotted lines). We clearly distinguish the emergence of three subshells in the entropy. The most entangled qubits are those in the lowest-energy orbital, $0{d_{5/2}}$, reaching almost the maximal value. These are followed by the $1{s_{1/2}}$ and the $0{d_{3/2}}$ states, which are correspondingly less entangled (and occupied). The entropies saturate to the shell-model value relatively quickly, within about 20 layers. We take this as an indication that ADAPT-VQE captures early on the most important correlations of the nucleus, which are subsequently refined by the variational process.

## Discussion

In this work, we provide a detailed framework for a quantum hardware implementation of ADAPT-VQE tailored to nuclear shell-model calculations. The algorithm requires as many qubits as the number of single-particle states, a relatively small number ($\approx 50$) even for valence spaces demanding currently unavailable classical computational resources. We benchmark our results with calculations using a circuit-free, regular matrix implementation of the algorithm.

Our simulations do not become stuck in local minima or barren plateaus. We find that the majority of the resources in the quantum circuit are dedicated to the construction of the parametrized ansatz wave function. Each additional parameter in the ansatz increases the circuit depth linearly with the number of qubits. In contrast, the preparation of the reference state and the implementation of the basis changes to measure Hamiltonian expectation values are comparatively small parts of the total circuit depth. We quantify (see “Methods” section) the number of circuits needed to measure energies in the different isotopes. Our proposed energy-measuring circuits are not substantially deeper than the corresponding circuit encoding the wave function.

**Figure 4:**
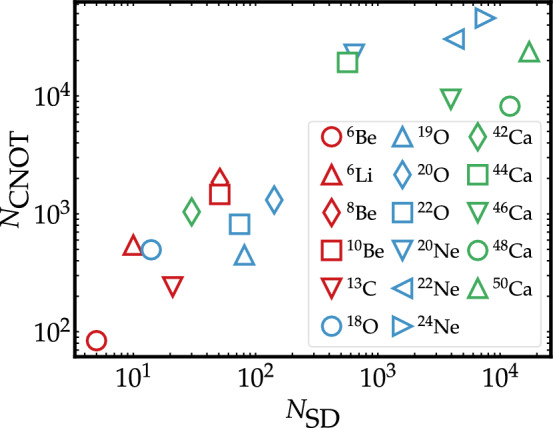
Correlation between number of CNOTs and Slater determinants. Total number of CNOTs $N_{\text {CNOT}}$ needed to obtain a ground-state relative energy error of $2\%$ as a function of the number of Slater determinants $N_{\text {SD}}$ in the many-body basis for all nuclei considered in this work. The observed trend does not indicate a quantum exponential advantage over classical methods.

We calculate the ground state of selected nuclei in the *p-*, *sd-* and *pf-*shell valence spaces, using up to 24 qubits. For all these systems, our simulations indicate that the relative error in the ground-state energy and the infidelity decrease exponentially as the number of layers in the ansatz increases (see Supplementary Information). While the number of parameters needed to reach a certain precision depends on the nucleus, our results indicate that at most 150 CNOT gates per ADAPT-VQE layer are necessary to get ground-state energies accurate at the percent level. This suggests that a circuit implementation of the shell model with ADAPT-VQE may be a suitable way forward for quantum computing simulations of nuclei. Nevertheless, the number of layers and CNOTs shown in Table 2 do not demonstrate an exponential quantum advantage^73^ with respect to the classical computation cost. This is indeed seen more clearly in Fig. 4, which shows the number of total CNOTs needed to obtain an energy relative error of 2%, as a function of the number of Slater determinants for all nuclei studied in this work. Figure 4 indicates that up to nuclear masses $A \simeq 50$ the number of CNOT gates scales roughly as the number of Slater determinants.

Our study opens several potential avenues for further exploration. First, different fermionic encodings may reduce the number of CNOT gates, which are subject to noise errors that can limit realistic implementations in quantum devices. A preliminary analysis using the Bravyi–Kitaev basis^58^ (instead of a JW transformation) suggests a $\approx 10\%$ reduction in the number of CNOT gates of ADAPT-VQE after 100 iterations in the *sd* and *pf* shells. Other options of fermionic mappings such as Gray code encoding^74,75^ should also be explored. Second, the present work is an ideal testbed for the implementation of quantum information tools for the study of nuclear structure. Our calculated single-particle state entropies reveal the entanglement structure of nuclei, in close analogy to the occupation probabilities of the orbitals obtained in classical diagonalization schemes. Other correlation measures, such as quantum discord^32,76,77^, will be the subject of future work. Furthermore, one should elucidate more clearly the sharp differences between the UCC and ADAPT ansatz VQEs. On the one hand, the choice of initial states, at the mean-field level^43,44^ or mixing many-body configurations, may improve the overall performance^36,59^ of the minimization process. On the other, understanding why the ordering in the choice of operators is so relevant may provide further insights into nuclear many-body correlations. A better understanding on these issues is key to find optimal algorithms and circuit designs for the nuclear shell model that avoid the exponential scaling of resources and can be realistically implemented in NISQ devices. We note that there are promising alternative algorithms for nuclear shell-model calculations based on the Lanczos method^78^.

## Methods

We simulate circuits for several *p*-, *sd*- and *pf*-shell nuclei using the statevector simulator qibo^79^, together with the qibojit package, which harnesses multi-core parallelization based on JIT (just-in-time) compilation and the numba compiler^80^. qibo has been found to be specially efficient when compared to other simulators for similar fermionic quantum-circuit simulations^81^. At each layer, we execute the quantum circuit to extract a statevector $|\psi _n\rangle$ of dimension $2^{N_{qb}}$. This extraction is limited by classical computer resources, which in turn provide stringent mass limits for our classical circuit simulations. For instance, simulating open-shell nuclei in the *pf* shell valence space, requires state-vectors with $2^{40}$ complex coefficients, demanding 8 TB of memory in single-precision format. When dealing with 20 or more qubits, we use GPUs and the cupy compiler^82^ to accelerate computations.

**Table 3:** Jordan–Wigner transformation for the main operators appearing in the Hamiltonian and in our ADAPT-VQE operator pool.

	Fermion operators	Qubit operators
$n_p$	$a_p^{\dag }a_p$	$\dfrac{1}{2} (1-Z_p)$
$h_{pqrs}$	$$\begin{array}{l} a_p^{\dag }a_q^{\dag }a_r a_s \\ \quad + a_r^{\dag }a_s^{\dag }a_p a_q\end{array}$$	$$\begin{array}{l} \dfrac{1}{8} P_{rs}^{pq}\, ( -X_p X_q X_r X_s + X_p X_q Y_r Y_s \\ \quad \qquad - X_p Y_q X_r Y_s- X_p Y_q Y_r X_s \\ \quad \qquad - Y_p Y_q Y_r Y_s + Y_p Y_q X_r X_s \\ \quad \qquad - Y_p X_q Y_r X_s - Y_p X_q X_r Y_s)\end{array}$$
$T_{rs}^{pq}$	$$\begin{array}{l} i(a_p^{\dag }a_q^{\dag }a_r a_s \\ \quad - a_r^{\dag }a_s^{\dag }a_p a_q)\end{array}$$	$$\begin{array}{l} \dfrac{1}{8} P_{rs}^{pq}\, ( -X_p Y_q Y_r Y_s - Y_p X_q Y_r Y_s \\ \quad \qquad + Y_p Y_q X_r Y_s + Y_p Y_q Y_r X_s \\ \quad \qquad +Y_p X_q X_r X_s + X_p Y_q X_r X_s \\ \quad \qquad - X_p X_q Y_r X_s - X_p X_q X_r Y_s)\end{array}$$
$h_{pq}$	$a_p^{\dag }a_q + a_q^{\dag }a_p$	$\dfrac{1}{2}\left( \prod _{n=p+1}^{q-1}Z_n\right) \left( X_p X_q+Y_q Y_p\right)$
$T_{pq}$	$i(a_p^{\dag }a_q - a_q^{\dag } a_p)$	$\dfrac{1}{2}\left( \prod _{n=p+1}^{q-1}Z_n\right) \left( Y_p X_q-X_q Y_p\right)$

Indices run over $p<q$ and $r<s$, assuming that all are different. If two indices are repeated, then $h_{pqpr}=-n_p h_{qr}$ and $T_{pq}^{pr}=n_p T_{qr}$, with $q<r$. We note that $h_{pqpq}=-2n_p n_q$ and $T_{pq}^{pq}=0$.

Next, we describe the five different stages^24^ of our VQE circuit design strategy.

### Mapping

We consider the JW mapping^58,83^, which transforms nucleonic creation and annihilation operators as

9 $$\begin{aligned} a_i^{\dag } = \left( \prod _{k=0}^{i-1}Z_k\right) \sigma ^-_i,~~ a_i = \left( \prod _{k=0}^{i-1} Z_k\right) \sigma ^+_i, \end{aligned}$$

where $\sigma ^\pm _j=\frac{1}{2}(X_j\pm i Y_j)$ and $X_j$, $Y_j$, $Z_j$ are the usual Pauli matrices applied to qubit *j*. Using these relations we can express any fermionic operator in terms of Pauli strings. Table 3 lists the expressions for the two types of (self-adjoint) terms appearing in the nuclear shell-model Hamiltonian $H_\mathrm{{eff}}$ in Eq. (1). We use an auxiliary operator

10 $$\begin{aligned} P_{rs}^{pq} \equiv \left( \prod _{m=p+1, m\notin [r,s]}^{q-1}Z_m\right) \left( \prod _{n=r+1,n\notin [p,q]}^{s-1}Z_n\right) . \end{aligned}$$

Table 3 also indicates the JW transformation for the pool operators $T_{rs}^{pq}$, and for single-excitation operators which appear when indices are repeated in either $h_{pqrs}$ or $T_{rs}^{pq}$. In this context, the most important features of an operator are the numbers and lengths of the Pauli strings they contain. These ultimately determine the efficiency in the circuit implementation of ADAPT-VQE. The two operators $h_{pqrs}$ and $T_{rs}^{pq}$ contain eight Pauli strings, each of length $L_{pqrs}=n_2+n_4-n_1-n_3+2$, where $n_1$, $n_2$, $n_3$ and $n_4$ are the indices *p*, *q*, *r* and *s* sorted in ascending order. For example, if $(p,q,r,s)=(2,8,5,7)$, then $(n_1,n_2,n_3,n_4)=(2,5,7,8)$ and $L_{2857}=6$. If two indices are repeated, the expressions simplify to $h_{pqpr}$ and $T_{pq}^{pr}$, as indicated in Table 3. These consist of two Pauli strings of length $L_{pqr}^{(1)}=r-q+1$ and two other strings of length $L_{pqr}^{(2)}=r-q+2$.

**Figure 5:**
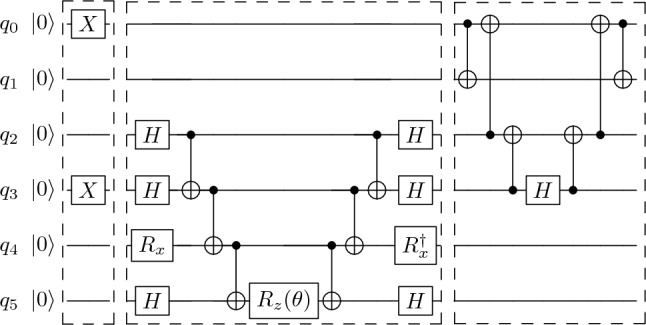
Examples of main circuit blocks, separated by dashed boxes, in ADAPT-VQE for the simulation of $^6$Be. Left: preparation of the reference state defined in Eqs. (11) and (12). Middle: implementation of $e^{-i\frac{\theta }{2} X_2X_3Y_4Z_5}$ using the CNOT staircase algorithm, one out of the many unitaries in the variational part of ADAPT-VQE. Right: circuit of the basis change $M_{0123}$ needed to diagonalize $h_{0123}$. The subcircuit in qubits $q_2$ and $q_3$ containing two CNOTs and a Hadamard gate *H* corresponds to the basis change $M_{23}$.

### Initial state preparation

To provide a minimal starting point to the simulations, we choose the lowest-energy Slater determinant as a reference state. Under the JW mapping, Slater determinants are mapped to the computational basis by flipping the qubits corresponding to the occupied orbitals using *X* gates. Considering for example the case of $^6$Be, an isotope in the *p* shell (panel (b) of Fig. 1) and for our interaction of choice, the lowest-energy Slater determinant is

11 $$\begin{aligned} |0,3\rangle = a_0^{\dag } a_3^{\dag }\,|\text{vac}\rangle , \end{aligned}$$

where $|\text{vac}\rangle$ is the vacuum state with no particles in the valence space. After a JW mapping, the state is translated into the computational basis as

12 $$\begin{aligned} |100100\rangle = X_0 X_3|000000\rangle . \end{aligned}$$

The leftmost block of Fig. 5 shows the corresponding circuit.

This choice of initial state preparation is minimal in terms of circuit resources: it has unit depth independently of the number of orbitals in the valence space and it does not involve any two-qubit gates. For a given valence neutron and proton number, $N_\text {CI}$ and $Z_\text {CI}$, finding the lowest energy Slater determinant requires at most $N_\text {SD}$ operations. This task can be performed relatively quickly in a classical computer, and is a one-off pre-processing overhead that we do not incorporate in the circuit resources discussed below.

**Figure 6:**
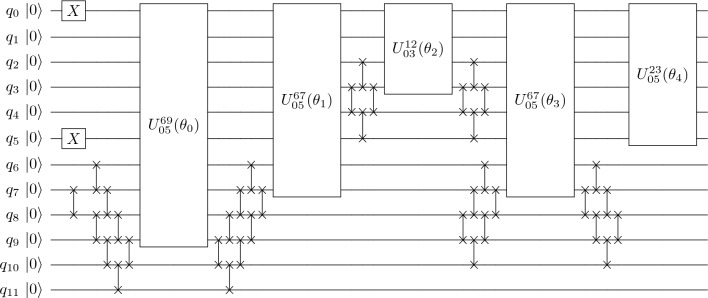
Circuit to prepare the $^{18}$O ground state. *X* gates prepare the reference state and FSWAP gates change the basis so that pool-operator exponentials act on adjacent qubits. Multiqubit gates in boxes are defined as $U_{rs}^{pq}(\theta )\equiv e^{i\theta T_{rs}^{pq}}$ and $\theta _0=-\,0.157263$, $\theta _1=-\,0.437238$, $\theta _2=0.604663$, $\theta _3=0.214431$, $\theta _4=-\,0.785469$.

### Variational optimization

The variational ansatz is parametrized as in Eq. (5), with pool operators $A_k=T_{rs}^{pq}$ given in Table 3 after the JW transformation. We convert the pool operators $T_{rs}^{pq}$ to Pauli strings using the OpenFermion package^84^, and for the circuits for the unitaries $e^{i\theta T_{rs}^{pq}}$ we follow the staircase algorithm of Fig. 5. In the simulated circuits we only use single-qubit and CNOT gates.

All Pauli strings in these sums commute with each other, so each term in $T_{rs}^{pq}$ can be exponentiated separately and there is no need for a Trotter-Suzuki approximation. This results in the expression

13 $$\begin{aligned} \begin{aligned} e^{i\theta T_{rs}^{pq}} =&e^{-i\theta ' P_{rs}^{pq} X_p Y_q Y_r Y_s} e^{-i\theta ' P_{rs}^{pq} Y_p X_q Y_r Y_s} e^{i\theta ' P_{rs}^{pq} Y_p Y_q X_r Y_s} e^{i\theta ' P_{rs}^{pq} Y_p Y_q Y_r X_s}\\&\times e^{i\theta ' P_{rs}^{pq} Y_p X_q X_r X_s} e^{i\theta ' P_{rs}^{pq} X_p Y_q X_r X_s} e^{-i\theta ' P_{rs}^{pq} X_p X_q Y_r X_s} e^{-i\theta ' P_{rs}^{pq} X_p X_q X_r Y_s }, \end{aligned} \end{aligned}$$

with $\theta '=\theta /8$ and $P_{rs}^{pq}$ given in Eq. (10). The exponential of a single Pauli string is particularly easy to implement with the staircase algorithm^85^. If the Pauli string contains only *Z* matrices, the circuit contains two cascades of CNOTs and a *Z* rotation, $R_z(\theta )\equiv e^{-i \frac{\theta }{2}Z}$, with $-\frac{\theta }{2}$ the coefficient multiplying the Pauli string. If the product contains an *X* or *Y* matrix, we apply a basis change in the corresponding qubit, namely $X=HZH$ and $Y=R_x^\dagger Z R_x$, where *H* is the Hadamard gate and $R_x$ the rotation $e^{-i \frac{\pi }{4}X}$. Figure 5 (middle) illustrates the procedure for the example implementation of $e^{-i\frac{\theta }{2} X_2X_3Y_4X_5}$. If $e^{i\theta T_{rs}^{pq}}$ acts on non-adjacent qubits, we implement a change of basis through fermionic SWAP (FSWAP) gates, so that only CNOTs applied to contiguous qubits are needed. The FSWAP exchanges states while maintaining the correct parity,

14 $$\begin{aligned} \text {FSWAP}=1+a_i^\dagger a_j+a_j^\dagger a_i-a_i^\dagger a_i-a_j^\dagger a_j. \end{aligned}$$

Using the staircase protocol, each parametrized layer $e^{i\theta T_{rs}^{pq}}$ requires $16\,(L_{pqrs}-1)$ CNOT gates, where $L_{pqrs}$ is the average length of the Pauli strings in the operator. $L_{pqrs}$ is bounded by the number of qubits $N_{qb}$, implying that the maximum number of CNOTs per ansatz layer is $16\,(N_{qb}-1)$ and that the depth per layer grows linearly with the number of single-particle states in the valence space. If qubits are linearly connected in hardware and non-adjacent qubit states are brought together with FSWAPs, the depth per layer has a total linear overhead. The precise overhead size depends on how qubits are arranged and connected to each other. However, it is bounded by $4(N_{qb}-4)$.

Let us provide an example illustrating the simplicity of the ADAPT-VQE circuit implementation. Obtaining the ground-state energy of simple nuclei only demands a few operators. As shown in Results, ADAPT-VQE simulations for $^{18}$O converge to an energy accuracy better than $10^{-6}$ with a five-layer ansatz, reading

$$\begin{aligned} |\psi _{ ^{18}\text{O}}\rangle = e^{i\theta _4 T^{05}_{23}}e^{i\theta _3 T^{05}_{9\,10}}e^{i\theta _2 T^{05}_{14}}e^{i\theta _1 T^{05}_{67}}e^{i\theta _0 T^{05}_{8\,11}}X_0X_5|0\rangle ^{\otimes 12}. \end{aligned}$$

Figure 6 shows the full circuit assuming one-dimensional connectivity between qubits, and gives the parameter values. Our algorithm includes the multiqubit operators $e^{i\theta T_{rs}^{pq}}$ involving CNOT gates acting on non-adjacent qubits when these are laid out in a one-dimensional array. We manipulate these operators to include only local two-qubit gates through a series of FSWAPs.

### Measurement

Once the ADAPT-VQE ansatz $|\psi _n \rangle$ is prepared in the quantum circuit at a given layer *n*, we measure the energy with the expectation value $\langle \psi _n|H_\mathrm{{eff}}|\psi _n\rangle$. To this end, we build a series of circuits that implement a change of basis to diagonalize separately each term of the Hamiltonian. The number of terms in the shell-model Hamiltonian scales with the number of qubits as $O(N_{qb}^4)$, but we find a much milder scaling of the circuit number with $N_{qb}$.

One-body (number) operators $n_i$ are diagonal and can be measured directly,

15 $$\begin{aligned} \langle \psi _n|n_i|\psi _n\rangle = \frac{1}{2} \langle \psi _n|1-Z_i|\psi _n\rangle = p_{1}^{(i)}, \end{aligned}$$

where $p_{1}^{(i)}$, the probability of measuring “1” in qubit *i*, can be extracted by measuring multiple times that qubit. Since all one-body operators commute with each other, we can measure all of them simultaneously. The two-body part of the Hamiltonian $h_{ijkl}$ can be divided into three kinds of terms depending on whether indices (*i*, *j*, *k*, *l*) are two, three, or four different integers. Local terms $h_{ijij}$ are the product of two number operators $n_i$ and $n_j$ and they can be measured simultaneously,

16 $$\begin{aligned} \langle \psi _n|h_{ijij}|\psi _n\rangle = -2\langle \psi _n|n_i n_j|\psi _n\rangle = -2 p_{11}^{(ij)}, \end{aligned}$$

with $p_{11}^{(ij)}$ the probability to measure “1” in qubits *i* and *j*. The non-diagonal parts of $h_{ijik}$ and $h_{ijkl}$ swap two states in the subspaces of qubits (*i*, *j*, *k*) and (*i*, *j*, *k*, *l*), respectively. These operators can be disentangled through series of CNOT gates and reduced to an *X* gate acting on a single qubit. The Pauli matrix *X* is then diagonalized with a Hadamard gate, $X = H Z H$. In turn, we diagonalize $h_{ijik}$ and $h_{ijkl}$ using $M_{jk}\equiv \,CX_{kj}\,H_k\,CX_{kj}$ and $M_{ijkl}\equiv \, CX_{ij}CX_{ki}CX_{lk} H_l CX_{lk} CX_{ki} CX_{ij}$, where $CX_{ij}$ represents a CNOT gate with control qubit *i* and target qubit *j*. The right block of Fig. 5 illustrates the corresponding circuit implementation. After diagonalization, assuming contiguous indices, the expectation values read

17 $$\begin{aligned} \begin{aligned} \langle \psi _n|h_{ijik}|\psi _n\rangle =&p_{101}^{(ijk)}-p_{110}^{(ijk)}, \end{aligned} \end{aligned}$$

and

18 $$\begin{aligned} \begin{aligned} \langle \psi _n|h_{ijkl}|\psi _n\rangle =&p_{1100}^{(ijkl)} - p_{0011}^{(ijkl)}, \end{aligned} \end{aligned}$$

with $p_{r_1 \cdots r_k}^{(q_1 \dots q_k)}$ being the probabilities of measuring results $r_1$ to $r_k$ in qubits $q_1$ to $q_k$ in the statevector where the basis changes have been applied. We refer to the Supplementary Information for a detailed derivation of Eqs. (17) and (18).

The changes of basis needed for measurements add, for any nucleus, an overhead of zero, two or six two-qubit gates depending on the Hamiltonian term measured. This represents a small fraction of the circuit depth and a constant scaling with the number of single-particle states in the valence space. We discuss in the Supplementary Information details regarding to the number of different measurement circuits required to measure the energy as well as the gradients of Eq. (7).

### Error mitigation

Finally, expectation values of the Hamiltonian computed using the algorithm described above are subject to statistical errors and quantum noise. The former scale as the inverse of the number of shots, $\sigma _E \propto \frac{1}{\sqrt{N_{s}}}$. In other words, given a target error in the energy accuracy $\varepsilon _{\langle H\rangle }$, the number of necessary shots scales as

19 $$\begin{aligned} N_s\propto \frac{1}{\varepsilon _{\langle H\rangle }^2}. \end{aligned}$$

The specific factor may be estimated simulating the measurement protocol. A straightforward and robust strategy to mitigate errors for ADAPT-VQE shell-model simulations is to use symmetry considerations and discard measurements that do not yield results consistent with the Fock basis of the simulated nucleus. Since the JW mapping identifies Fock and computational states, this amounts to excluding all states with different number of measured “1”s than nucleons in the valence space. Likewise, one should also ignore states with measured “1”s distributed in a set of qubits corresponding to a different angular momentum or isospin than the simulated nucleus. This protocol should be particularly effective in mitigating single bit-flip errors, which effectively create or destroy nucleons, as well as multiple bit-flip errors which do not preserve either nucleon number, angular momentum or isospin. These simple but robust strategies may be key in future implementations of this method on NISQ devices.

## Supplementary Information

Supplementary Information.

Supplementary Figure 1.

Supplementary Figure 2.

Supplementary Figure 3.

## Data Availability

The data that support the findings of this study are available within the paper and its Supplementary Information. Any additional information is available from the corresponding authors upon request.
