# Supplementary material for: Nuclear shell-model simulation in digital quantum computers
Source: Sci Rep. 2023 Jul 29;13:12291. doi: 10.1038/s41598-023-39263-7 (PMC10387092; doi:10.1038/s41598-023-39263-7)
Supplement: Supplementary file 1 — Supplementary Information. [file 41598_2023_39263_MOESM1_ESM.pdf]

Supplementary information on

Nuclear shell-model simulation in digital quantum computers

Axel Pérez-Obiol[1*], Antonio M. Romero[2,3*,], Javier Menéndez[2,3], Arnau Rios[2,3], Artur García-Sáez[1] & Bruno Juliá-Díaz[2,3]

[1]Barcelona Supercomputing Center, 08034 Barcelona, Spain. [2]Departament de Física Quàntica i Astrofísica (FQA), Universitat de Barcelona (UB), c. Martí i Franqués, 1, 08028 Barcelona, Spain. [3]Institut de Ciències del Cosmos (ICCUB), Universitat de Barcelona (UB), c. Martí i Franqués, 1, 08028 Barcelona, Spain. [4]Qilimanjaro Quantum Tech, 08007 Barcelona, Spain. [*]contributed equally. corresponding authors: a.marquez.romero@fqa.ub.edu, axel.perezobiol@bsc.es

Circuit design strategy

Number of different measurement circuits

Here we discuss the number of different measurement circuits that are necessary to compute expectation values of the energy as well as of the products of operators required in the gradient calculations. Local terms n_i and h_{ijij} can be measured simultaneously. We analyze and optimize the number of different circuits needed to measure the expectation value of the non-local part of H_{eff}, h_{ijki} and h_{ijkl}, for the p, sd and pf shell valence spaces.

All terms $h_{ijki} = -n_i(a_j^\dagger a_k + a_k^\dagger a_j)$ with the same hopping (same indices j, k) and different local terms n_i can be measured simultaneously since they commute, $[h_{ijik}, h_{i'ji'k}] = 0$. The local part of h_{ijik} conserves the third components of the angular momentum and isospin, m and t_z, implying that the complementary hopping term involves only indices in the same vertical axis in the panel (b) diagram of Fig. 1 in the main text. For example, considering the sd shell with only neutrons, this amounts to a total of eight terms:

$$\{(j,k)\} = \{(1,8),(2,6),(6,9),(2,9),(3,7),(7,10),(3,10),(4,11)\}. \tag{1}$$

The number of different circuits needed to measure all h_{ijki} terms is then equivalent to the number of different m- and t_z-conserving single-excitation operators in the shell. This scales, in the worst case, as $O(N_{qb}^2)$, representing a relatively small number of circuits. Each term is diagonalized with the circuit $M_{jk} = CX_{kj}H_kCX_{kj}$, which for continguous indices, $k = j+1$, results in the operator $|101\rangle\langle 101| - |110\rangle\langle 110|$, where the indices (i,j,k) have been omitted. Therefore, $\langle h_{ijik}\rangle = p_{101}^{(ijk)} - p_{110}^{(ijk)}$, with $p_{101}^{(ijk)}$ and $p_{110}^{(ijk)}$ the probabilities of measuring 101 and 110 in qubits (i,j,k) after the change of basis.

The double-hopping terms h_{ijkl} that involve different sets of orbitals (i, j, k, l) also commute and can be measured with the same circuit. Given a group of self-commuting terms, products of Zs of one or more terms h_{ijkl} appearing in the JW mapping may overlap with the indices of another term $h_{i'j'k'l'}$ in the group. A product of an even number of overlapping Zs, for example $P_{\text{even}} = Z_{i'}Z_{j'}$, commutes with $M_{i'j'k'l'}$ and the same circuit M_{ijkl} can be used for both. If there is a product of an odd number of overlapping Zs, P_{odd}, then $[P_{odd}, M_{i'j'k'l'}] \neq 0$ and all the different h_{ijkl} operators need to be diagonalized simultaneously. Some terms that share two indices also commute, but for simplicity we do not group them into the same measurement.

Simultaneous diagonalization of double-hopping terms with different indices

Measuring the expected value of the Hamiltonian requires then a simultaneous diagonalization of each term h_{ijkl} with different values for the indices (i, j, k, l). These operators consist of the product $h_{ijkl} = P_{ij}^{kl} O_{ijkl}$, where P_{ij}^{kl} is a diagonal Pauli string containing only Zs and O_{ijkl} is the non-diagonal part,

$$\begin{aligned} O_{ijkl} \equiv & \left(\sigma_i^- \sigma_j^- \sigma_k^+ \sigma_l^+ + \sigma_k^- \sigma_l^- \sigma_i^+ \sigma_j^+\right) \\ = & \frac{1}{8}(X_iX_jX_kX_l - X_iX_jY_kY_l + X_iY_jX_kY_l \\ & + X_iY_jY_kX_l + Y_iY_jY_kY_l - Y_iY_jX_kX_l \\ & + Y_iX_jY_kX_l + Y_iX_jX_kY_l), \\ = & |0011\rangle\langle 1100| + |1100\rangle\langle 0011|, \end{aligned} \tag{2}$$

where in the last line the indices (i,j,k,l) have been omitted, see Table 1 in the main text. To diagonalize a single term h_{ijkl} we use the change of basis $M_{ijkl} \equiv CX_{ij}CX_{ki}CX_{lk}H_lCX_{lk}CX_{ki}CX_{ij}$, such that

$$M_{ijkl}^\dagger O_{ijkl} M_{ijkl} \equiv D_{ijkl} = |1100\rangle\langle 1100| - |0011\rangle\langle 0011|. \tag{3}$$

For contiguous indices, $j = i+1$, $l = k+1$, then $P_{ij}^{kl} = 1$, and we have $\langle h_{ijkl}\rangle = p_{1100}^{(ijkl)} - p_{0011}^{(ijkl)}$, dependent on the probabilities of measuring 1100 and 0011 in qubits (i, j, k, l) after applying the change of basis, as stated in Eq. (18) in the main text. In the general case, $j > i+1$, $l > k+1$, and $P_{ij}^{kl} \neq 1$, the expected value needs to account for the product of Z matrices. For example, considering $\langle Z_q\rangle = p_0^{(q)} - p_1^{(q)}$ and $\langle Z_qZ_r\rangle = p_{00}^{(qr)} - p_{01}^{(qr)} - p_{10}^{(qr)} + p_{11}^{(qr)}$,

$$\begin{aligned} \langle Z_q O_{ijkl}\rangle = & [p_{01100}^{(qijkl)} - p_{00011}^{(qijkl)}] - [p_{11100}^{(qijkl)} - p_{10011}^{(qijkl)}] \\ \langle Z_qZ_r O_{ijkl}\rangle = & [p_{001100}^{(qrijkl)} - p_{000011}^{(qrijkl)}] - [p_{011100}^{(qrijkl)} - p_{010011}^{(qrijkl)}] - [p_{101100}^{(qrijkl)} - p_{100011}^{(qrijkl)}] + [p_{111100}^{(qrijkl)} - p_{110011}^{(qrijkl)}]. \end{aligned} \tag{4}$$

In the case where two terms h_{ijkl}, $h_{i'j'k'l'}$ are simultaneously diagonalized, the indices from the product of Z matrices in each term might overlap. If there is an even number of overlapping Z matrices, P_{ij}^{kl} commutes with $M_{i'j'k'l'}$ and the same circuit

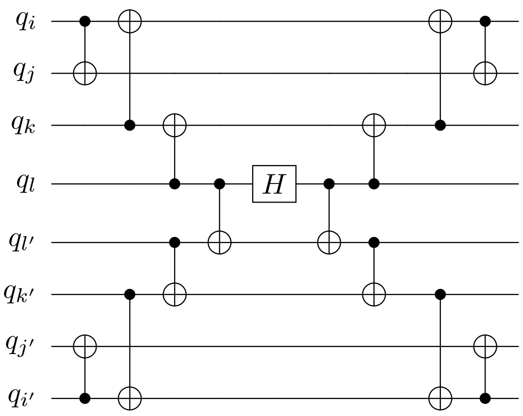

Figure 1. Quantum circuit to implement the change of basis to diagonalize $Z_{l'}O_{ijkl}Z_lO_{i'j'k'l'}$ for double-hopping terms.

$M_{i'j'k'l'}$ to diagonalize $h_{i'j'k'l'}$ can be used, since P^{kl}_{ij} can be factored out. The same holds for M_{ijkl}. For example, if there are two overlapping Zs,

$$\begin{aligned} &M^\dagger_{ijkl}M^\dagger_{i'j'k'l'}\left(Z_{i'}Z_{j'}O_{ijkl}\right)\left(Z_iZ_jO_{i'j'k'l'}\right)M_{ijkl}M_{i'j'k'l'} \\ &=\left(M^\dagger_{ijkl}O_{ijkl}M_{ijkl}\right)\left(M^\dagger_{i'j'k'l'}O_{i'j'k'l'}M_{i'j'k'l'}\right)Z_iZ_jZ_{i'}Z_{j'} \\ &=D_{ijkl}D_{i'j'k'l'}Z_iZ_jZ_{i'}Z_{j'}, \end{aligned} \tag{5}$$

with D_{ijkl} and $D_{i'j'k'l'}$ the corresponding diagonal operators. If P^{kl}_{ij} contains a product of three Zs overlapping with (i', j', k', l'), then two can be factored out so that the problem is reduced to simultaneously diagonalizing operators $Z_{l'}O_{ijkl}$ and $Z_lO_{i'j'k'l'}$.

In practice, we only need to build new circuits that diagonalize a 2-qubit subspace, instead of the full 8-qubit space. The non-diagonal part O_{ijkl} exchanges the states $|0011\rangle$ and $|1100\rangle$, effectively operating in this two-state subspace through an X gate. The circuit in the right dashed box of Fig. 2 in the main text can be interpreted as a three-step protocol. First, a change of basis through a set of CNOT gates such that X operates only in the last qubit; second, a Hadamard gate acting on that qubit to diagonalize X, $HXH=Z$; and third, the inverse sequence of CNOTs to switch back to the original basis. If one term has an overlapping Z, then instead of the Hadamard gate acting separately on each 4-qubit circuit, we need to diagonalize the corresponding 2-qubit space. For example, if we want to measure $Z_{i'}O_{ijkl}$ and $Z_iO_{i'j'k'l'}$ with the same circuit, we need to diagonalize $X_lZ_{l'}$ and $Z_lX_{l'}$, and embed the corresponding circuit, $CX_{ll'}H_{l'}CX_{ll'}$, within the change of basis, see Fig. 1.

Circuits to diagonalize products of Hamiltonian and pool operators

In order to measure gradients using Eq. (7) in the main text, we need to compute expected values of $h_{ijkl}T^{rs}_{pq}$. Similarly to O_{ijkl}, this operator effectively swaps two states in the computational basis,

$$\begin{aligned} h_{ijkl}T^{rs}_{pq} = i|11001100\rangle\langle 00110011| - i|00110011\rangle\langle 11001100| \\ + i|00111100\rangle\langle 11000011| - i|11000011\rangle\langle 00111100|, \end{aligned} \tag{6}$$

where we have assumed $P^{kl}_{ij} = P^{rs}_{pq} = 1$. This operator can be disentangled through a series of CNOT gates up to the 2-qubit operator X_iY_p, which is then diagonalized with the basis change $CX_{ip}R_{xi}CX_{ip}$. Figure. 2 illustrates the full circuit to diagonalize $h_{ijkl}T^{rs}_{pq}$.

Discussion on the complete simulation set

We choose the Cohen-Kurath interaction[1] in the p shell, USDB[2] in the sd shell and KB3G in the pf shell[3]. Explicit three-nucleon interactions are typically neglected because their leading effects can be written as an effective two-body term[4,5].

Figure 3 shows the dependence on the number of ansatz layers of the energy error $\varepsilon_{\rm E}$ (top panels), infidelities I (second-row panels), number of CNOTs $N_{\rm CNOT}$ (third-row panels) and number of cost-function calls used by the classical optimizer $N_{\rm fc}$ (bottom panels) for all nuclei considered in this work. The iterative evolution shown by Fig. 3 presents similar features to Figs. 4 and 5 of the main text, where results are shown only for selected nuclei.

The first column of Fig. 3 indicates that all nuclei in the p shell are relatively straightforward to implement. They all converge quickly, reaching a relative ground-state energy error $\varepsilon_{\rm E} < 10^{-3}$ with only a dozen layers. Only ^{8}Be and ^{10}Be, with 2

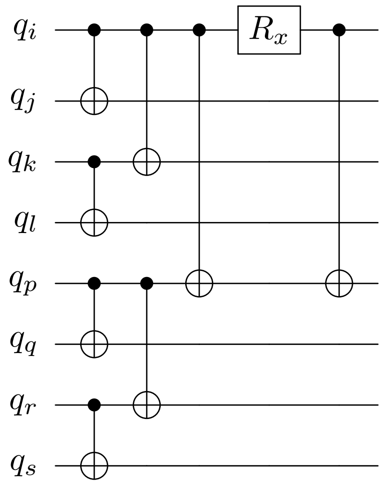

Figure 2. Quantum circuit M_{ijkl}^{pqrs} to diagonalize $h_{ijkl}T_{pq}^{rs}$ when all eight indices are different. The corresponding expectation value, $\langle\psi_n|h_{ijkl}T_{pq}^{rs}|\psi_n\rangle = -p_{00100010} + p_{00101010} - p_{10100010} + p_{10101010}$, depends on p_m, the probabilities of measuring m in the corresponding qubits (i, j, k, l, p, q, r, s) in the statevector $M_{ijkl}^{pqrs}|\psi_n\rangle$.

valence protons and 2 and 4 valence neutrons, respectively, require circuit architectures with ≈ 50 layers in order to capture their open-shell correlations, converging to a precision below $\varepsilon_{\rm E} = 10^{-6}$. We show $N_{\rm CNOT}$ and $N_{\rm fc}$ only up to this point, since this is the accuracy threshold of the classical minimizer. For all cases, the number of CNOT gates increases smoothly with numbers between 65 and 85 gates per layer. Thus, the implementation of p-shell nuclei in quantum circuits is promising in terms of both width (number of qubits, $N_{qb} = 12$ in this case) and depth (number of total CNOTs).

Using a single Slater determinant as a reference state is usually enough for the adaptive iterative procedure to reach the ground-state energy and wavefunction exponentially by increasing the number of parameters. In some cases, for particularly correlated systems, the initial state may be closer in structure to an excited state than the ground state, and one may land into the local minimum corresponding to the excited state. The only such situation we encountered is ^{6}Li, where a simple change of reference state was sufficient to converge into the ground state. We also note that ^{6}Be is represented in the figure, but it converges in only 2 layers.

The second column of Fig. 3 shows results for oxygen isotopes (with no valence protons) and neon (two valence protons) in the sd shell, studied with circuits of $N_{qb} = 12$ and 24 qubits, respectively. We observe a stark difference in the simulation of both isotopic chains: the adaptive procedure —starting from a single Slater determinant reference state— needs significantly more layers to capture the many-body correlations present in open-shell neon isotopes. This is due to the relatively large many-body basis dimension of these neon isotopes $\dim_{\rm mb} \approx 10^4 - 10^5$ (see the right panel of Fig. 1 in the main text). Nevertheless, the number of CNOT gates scales at most polynomially with the number of layers, with between 90 and 100 gates per layer for oxygen and between 110 and 150 for neon isotopes. This relatively mild non-exponential scaling is promising toward the implementation of ADAPT-VQE in NISQ devices. The bottom panel shows that the number of calls to the cost function used by the classical optimizer at a given iteration is similar for sd- and p-shell nuclei. This suggests that there is no bottleneck in resources associated to the classical optimizer.

Finally, the third column of Fig. 3 presents the results for calcium isotopes (with no valence protons) in the pf shell, using circuits with $N_{\rm qb} = 20$ qubits. The first isotope, ^{42}Ca, convergences extremely quickly, within 10 layers. In contrast, calcium isotopes with more than 2 valence neutrons result in a slow convergence, similar to the one for neon isotopes. Again, these calcium isotopes have $\dim_{\rm mb} \approx 10^4 - 10^5$, and the algorithm needs more updates of the wavefunction to capture the strong correlations in their ground states. We find the slowest convergence for ^{44}Ca, a midshell isotope between the closed-shell ^{40}Ca and ^{48}Ca. Likewise, the infidelity of ^{44}Ca seems to stall around $I \approx 3 \times 10^{-2}$ and even the number of CNOT gates per layer grows beyond the range found for the rest of isotopes. This suggests that a different choice of reference state, involving more many-body basis states, may be required for a faster convergence and, as a result, a reduction in quantum resources. In contrast, we find again that the number of cost-function calls for all calcium isotopes follows a similar trend to the p- and sd-shell nuclei.

References

1. Cohen, S. & Kurath, D. Effective interactions for the 1p shell. *Nucl. Phys.* **73**, 1–24 (1965).

2. Brown, B. A. & Richter, W. A. New "USD" Hamiltonians for the *sd* shell. *Phys. Rev. C* **74**, 034315, DOI: 10.1103/PhysRevC.74.034315 (2006).

3. Poves, A., Sánchez-Solano, J., Caurier, E. & Nowacki, F. Shell model study of the isobaric chains A=50, A=51 and A=52. *Nucl. Phys. A* **694**, 157–198, DOI: https://doi.org/10.1016/S0375-9474(01)00967-8 (2001).

4. Carbone, A., Cipollone, A., Barbieri, C., Rios, A. & Polls, A. Self-consistent Green's functions formalism with three-body interactions. *Phys. Rev. C* **88**, 054326, DOI: 10.1103/PhysRevC.88.054326 (2013).

5. Hebeler, K. Three-nucleon forces: Implementation and applications to atomic nuclei and dense matter. *Phys. Reports* **890**, 1–116, DOI: 10.1016/j.physrep.2020.08.009 (2021). arxiv:2002.09548.

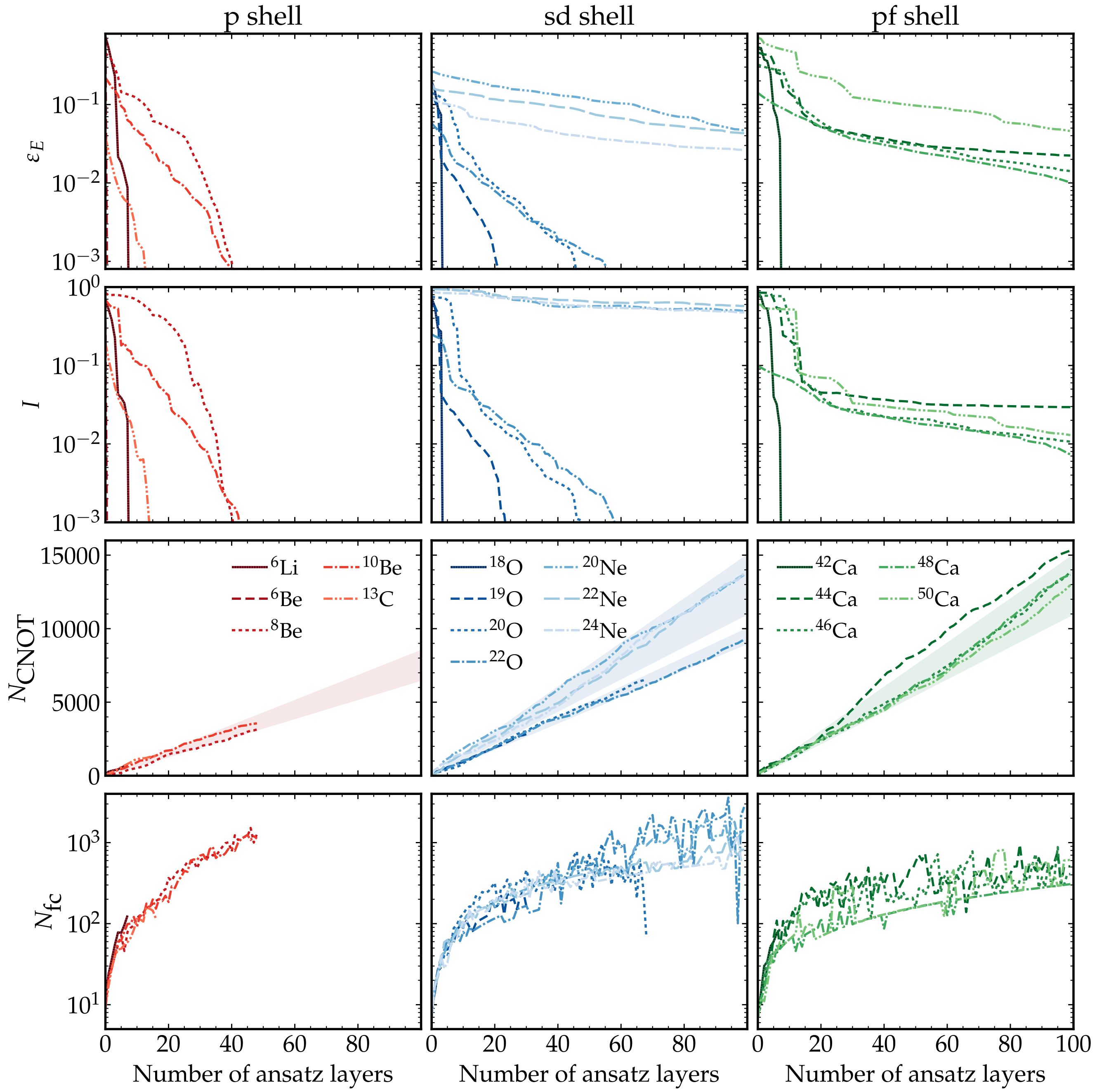

Figure 3. Evolution of the relative error for the ground-state energy, ε_{E}, (top row), infidelity I (second row), number of CNOT gates in the ansatz circuit N_{CNOT} (third row) and number of cost-function calls N_{fc} in the classical optimizer (bottom row) as a function of the number of ansatz layers for simulations of all p-shell (first column), sd-shell (second column) and pf-shell (third column) nuclei considered in this work. The bands in the number of CNOT gates panels are meant to guide the eye and correspond to lower (upper) limits of CNOT gates per layer of 65 (85) in p-shell nuclei, 90 (100) in oxygen isotopes and 110 (150) in both neon and calcium isotopes. The number of CNOT gates increases polynomially even in least favorable cases of convergence of ^{44}Ca and ^{24}Ne. The relative energy error and infidelities follow analogous trends during the iterative process. This indicates that the algorithm captures the correlations in the nuclear wavefunctions. The number of calls to the cost-function for the classical optimization presents a similar trend for all nuclei, mildly increasing on average with the number of layers.
