# Supplementary figures and images for: Nuclear shell-model simulation in digital quantum computers
Source: Sci Rep. 2023 Jul 29;13:12291. doi: 10.1038/s41598-023-39263-7 (PMC10387092; doi:10.1038/s41598-023-39263-7)

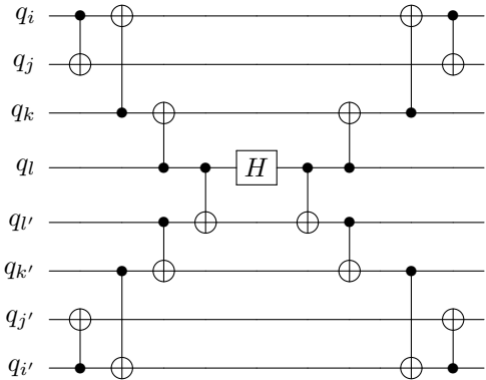

Supplement: Supplementary file 2 — Supplementary Figure 1. [file 41598_2023_39263_MOESM2_ESM.pdf]

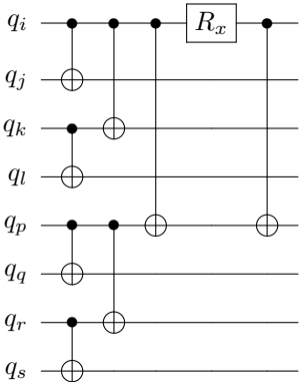

Supplement: Supplementary file 3 — Supplementary Figure 2. [file 41598_2023_39263_MOESM3_ESM.pdf]

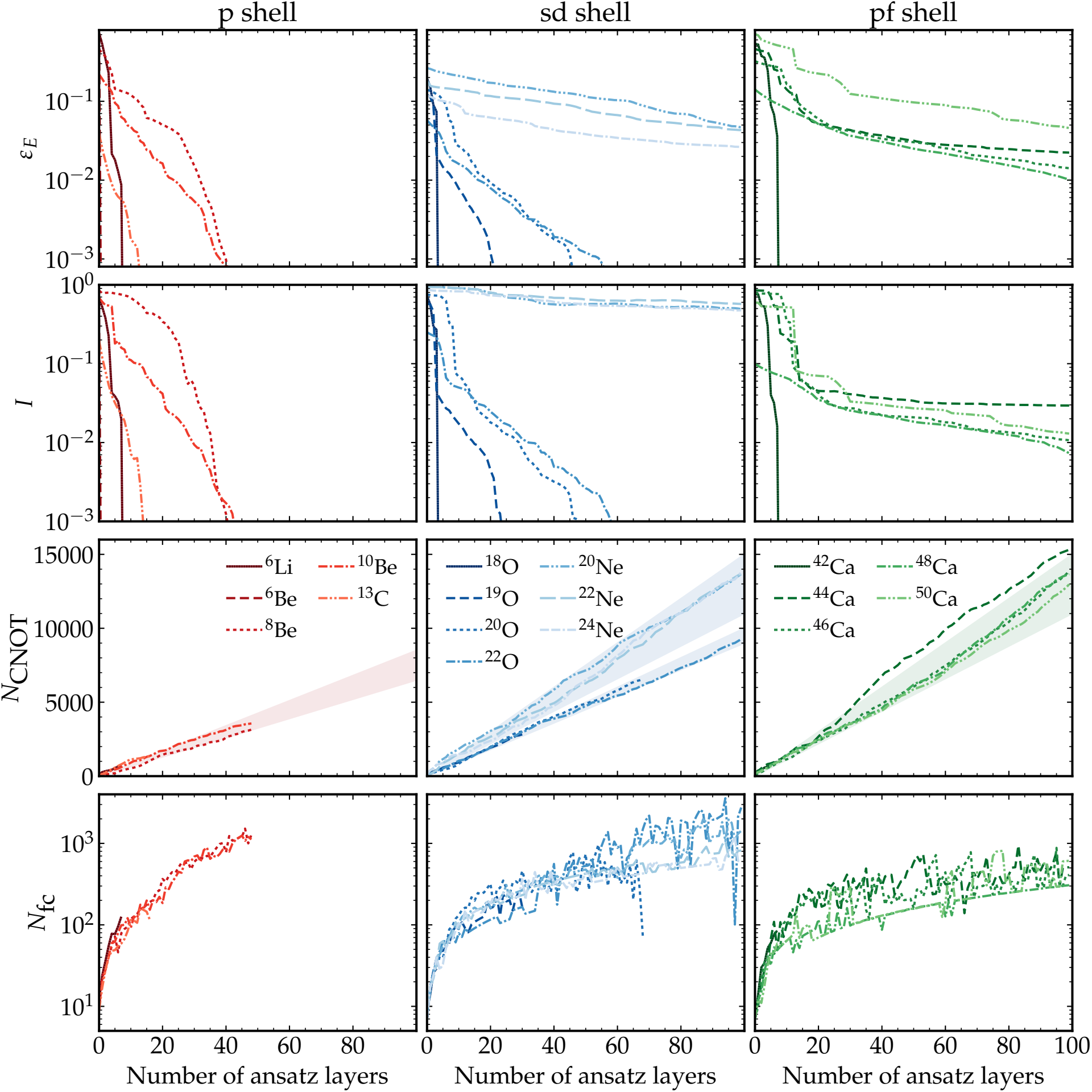

Supplement: Supplementary file 4 — Supplementary Figure 3. [file 41598_2023_39263_MOESM4_ESM.pdf]
